# Temporal and spatial evolution and obstacle diagnosis of resource and environment carrying capacity in the Loess Plateau

**DOI:** 10.1371/journal.pone.0256334

**Published:** 2021-08-18

**Authors:** Huan Huang, Rui Wang, Jue Wang, Jixing Chai, Yi Xiao

**Affiliations:** 1 Business School, Chengdu University of Technology, Chengdu, Sichuan, China; 2 School of Management Science, Chengdu University of Technology, Chengdu, Sichuan, China; Institute for Advanced Sustainability Studies, GERMANY

## Abstract

Natural resources are scarce in the Loess Plateau, and the ecological environment is fragile. Sustainable development requires special attention to resource and environmental carrying capacity (RECC). This study selected 24 representative cities in five natural areas of the Loess Plateau; used the entropy-weight-based TOPSIS method to evaluate and analyze the RECC of each city and region from 2013 to 2018; established a diagnosis model to identify the obstacle factors restricting the improvement of RECC; and constructed the theoretical framework of the RECC system mechanism. The results show that the RECC of the Loess Plateau is increasing in general but is relatively small. The environmental and social subsystems have the highest and lowest carrying capacities, respectively. There is an evident contradiction between economic development and the environment. Population density, investment in technological innovation, per capita sown area, and per capita water resources are the main obstacles affecting the improvement of RECC in the Loess Plateau. Such evaluations and diagnoses can support ecological civilization and sustainable development.

## Introduction

At present, global sustainable development faces challenges such as population growth [1], climate change [2, 3], over-exploitation of resources [4], and resource depletion [5]. Human needs are continually increasing, but the natural resources available for human use are limited. Rapid social and economic development often come at the expense of natural resources and the environment, resulting in increasingly severe resource and environmental problems, which will restrict the further development of human society. Dealing with resource and environmental issues, and realizing the harmonious co-generation of humans and nature is a major problem that needs to be solved urgently. The antagonistic relationship between humans and nature has led people to enter a reflective development model. From *"The Limits of Growth"* in the 1970s [6] to the United Nations Conference on Environment and Development in the 1990s, people gradually began to pursue sustainable development.

China is currently under tremendous pressure from economic growth, energy consumption, and environmental crisis [7]. Greenhouse gas emissions, energy shortages, and reduced environmental capacity restrict sustainable development in China [8]. With rapid economic development, the contradiction between the social economy and the ecological environment has become increasingly prominent in most resource-based cities in China. A coordinated development between the two is significant for the sustainable development of such cities [9]. Evaluating the resource and environmental carrying capacity (RECC) of a region provides solutions for realizing the harmonious coexistence of humans and nature. Analyzing the relationship between regional resources, the environment, and human activities plays a vital role in regional sustainable development [10].

The Loess Plateau is located in western China and is one of the four largest plateaus in China. It is the most concentrated and largest loess region worldwide, with a total area of 640,000 km^2^, and its scope mainly includes most or a part of the administrative regions of Shanxi, Inner Mongolia, Henan, Shaanxi, Gansu, Qinghai, and Ningxia. The Loess Plateau is the birthplace of the Chinese civilization. During the Spring and Autumn Period, and the Warring States Period (770 B.C.-221 B.C.) [11, 12], the forest coverage rate exceeded 50%. Before the Sui dynasty (A.D. 581–618), the Yellow River flooded only 1.1 times per century [13]. The vegetation status in the Loess Plateau has changed significantly. Because of unreasonable human production activities and excessive development, ignoring the destruction and deterioration of the environment, the grassland boundary of the Loess Plateau has moved south, the forest area is shrinking gradually, soil erosion is severe, and it is now a barren land [14, 15]. This is because the extraction and transport of resources, driven by societal and economic pressures, influence biodiversity and redefine the ecological status of the ecosystem [16]. Paying attention to RECC analysis, we can understand the current status and development trend of regional carrying capacity, which is of great significance for promoting ecological balance, achieving harmony between humankind and land, and promoting the coordinated development of resources and the environment [17].

As a typical ecologically fragile area in China, the Loess Plateau has a high annual average soil erosion rate, which varies from 2,000–20,000 t/km^2^/year, affecting approximately 70%–80% of the total area. The annual soil loss is 2.4–2.5 × l0^9^ tons [18]. The region has a relatively low annual average rainfall (∼400 mm) [19]. In the year 2000, woods (i.e. forests and shrubs) and grasses in the Loess Plateau region covered areas of 77.3 and 252.8 thousand km^2^, respectively [20]. If shrubs and open forests are excluded, the forest cover of the region is only 6.5% [12]. Continuing the model of economic development at the expense of the ecological environment will seriously damage the ecological environment and constrain coordinated development. Exploring whether socioeconomic development is in harmony with the resources and environment, and evaluating and analyzing the RECC is of great significance to the construction of local ecological civilization and sustainable development. Resource and social economy are complex and dynamic systems. Scholars have comprehensively used different methods for different research objects and have constantly improved and innovated the research methods of RECC.

By combining human activities with resources and the environment in carrying capacity analysis, this study constructs an RECC evaluation system, which includes social, economic, resource, and environmental factors. It discusses the resource and environment carrying levels of the representative region of the Loess Plateau. This will supplement existing research in the following ways.

1. The RECC of the Loess Plateau urban agglomeration is comprehensively evaluated from multiple dimensions. RECC is classified by combining each subsystem. Each subsystem is evaluated independently and comprehensively, which helps researchers assess the carrying level of each indicator in the Loess region. It also identifies the obstacle factors of RECC in an urban agglomeration and explores the greatest resistance to the improvement of RECC.
2. From a regional perspective, based on the inventory of resource-based cities in China, 24 representative cities, including provincial capitals, and small and medium-sized cities in five natural regions of the Loess Plateau, are selected as research objects. Studying the RECC of these cities can help understand the urbanization process of some cities in Northwest China and establish early warning mechanisms in the Loess Plateau and other ecologically vulnerable areas.
3. Taking the Loess Plateau as an example, this article puts forward theoretical suggestions for sustainable development and ecological civilization construction in ecologically fragile areas. It establishes the conceptual framework of the RECC influence mechanism to further explain the process of regional ecological development.

Section Two of the paper illustrates the RECC interactive framework; Section Three presents the research methods and data sources; Section Four presents the RECC evaluation results of representative cities and regions in the Loess Plateau, and discusses the obstacle factors for improving RECC; Section Five discusses the deficiencies of this study and the possibilities of future research, and further proposes the impact mechanism of RECC; and Section Six summarizes the findings of the research and presents some policy suggestions.

## Interactive framework of RECC

The evaluation of RECC is mainly based on the theory of resource scarcity [21], the theory of growth limit [6], the theory of harmony between humankind and nature [22], and the theory of sustainable development [23]. There is abundant research on RECC, which covers water resources carrying capacity [24], land resources carrying capacity [25, 26], atmospheric environment carrying capacity [27], and urban resources and environment carrying capacity [28] in different countries and regions. The results reveal that most countries consumed too many resources, thereby decreasing the overall global sustainability of natural resources that sustain the human society [29]. After continuously enriching the definition of RECC, scholars have begun to study methods and models. At present, the main evaluation methods for resource and environmental sustainability are environmental impact assessment [30], ecological footprint method [31], emergy analysis [32, 33], and cluster analysis [34, 35]. The main methods for determining the weight of the RECC index system are entropy method and analytic hierarchy process [36–38]. The entropy-weight-based TOPSIS method has high credibility and accuracy, and avoids the subjectivity of weight determination [39]. Scholars have mainly used the fuzzy evaluation method [40], system dynamics model [41], logistic growth model [42], and state-space model [43] to build their assessment models. Artificial neural network models [44, 45] and dynamic modeling [46] were used for the prediction and simulation of RECC.

**Fig 1:**
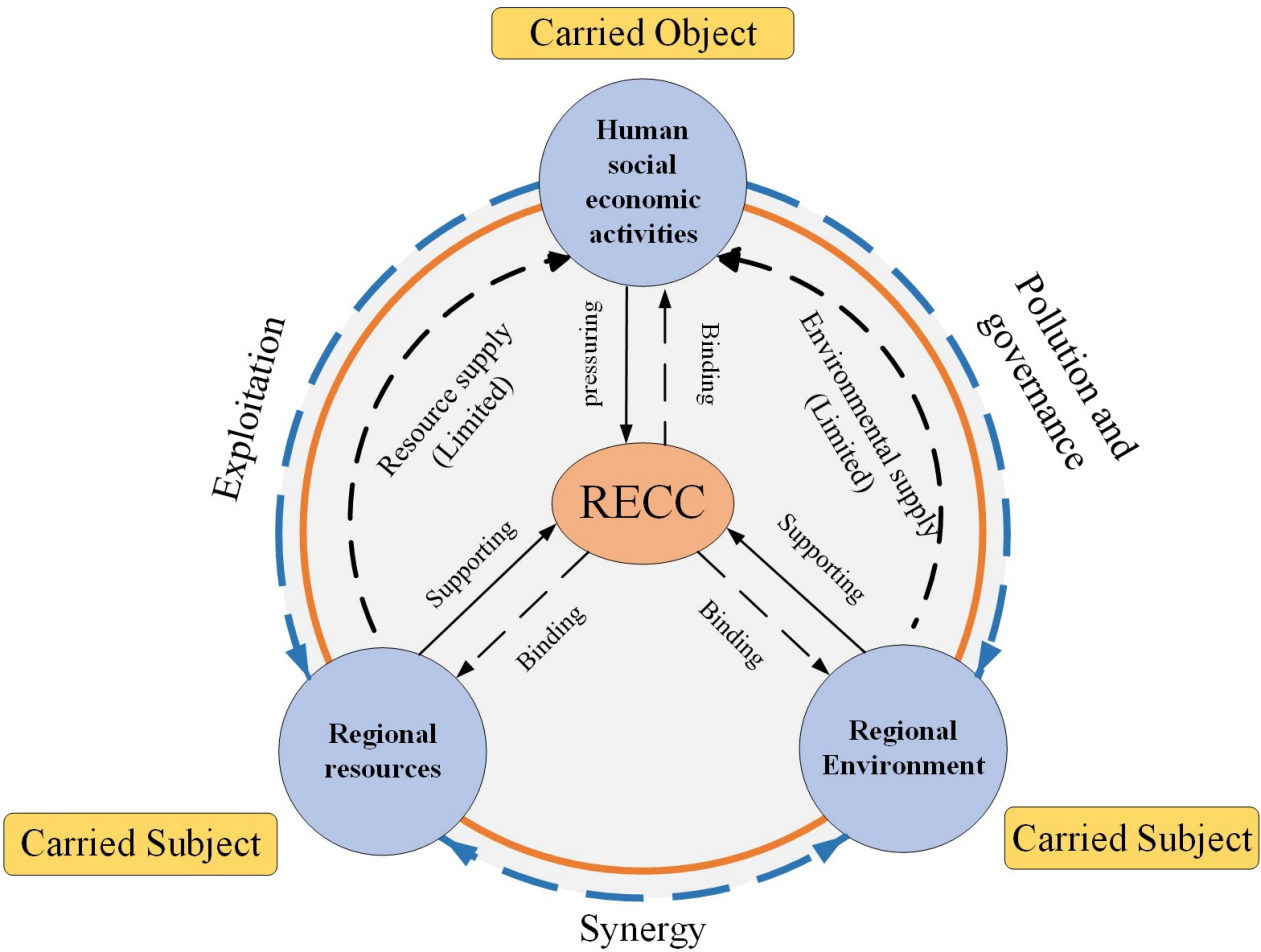
Interaction between the influencing factors of resource and environmental carrying capacity.

After more than 100 years of development, RECC has developed from single-element research on land resources, water resources, and ecological resource carrying capacity to comprehensive multi-element research involving resources, the environment, and ecology. Furthermore, RECC is considered to be the extension and development of ecological carrying capacity, resource carrying capacity, and environmental carrying capacity. RECC is mainly influenced by regional resources, the environment, and human activities. Regional resources and environment, as the carried subject, play a supporting role in RECC, while human social and economic activities endow RECC with bearing pressure. Generally speaking, regional resources and the environment provide resources and environmental supplies for human activities, while human social and economic activities will, in turn, exploit resources and pollute the environment. The coordination among the three is the key to influencing the RECC level. Fig 1 illustrates the interactive framework of the influencing factors of RECC.

## Research methods and data sources

### Overview of the research area

**Fig 2:**
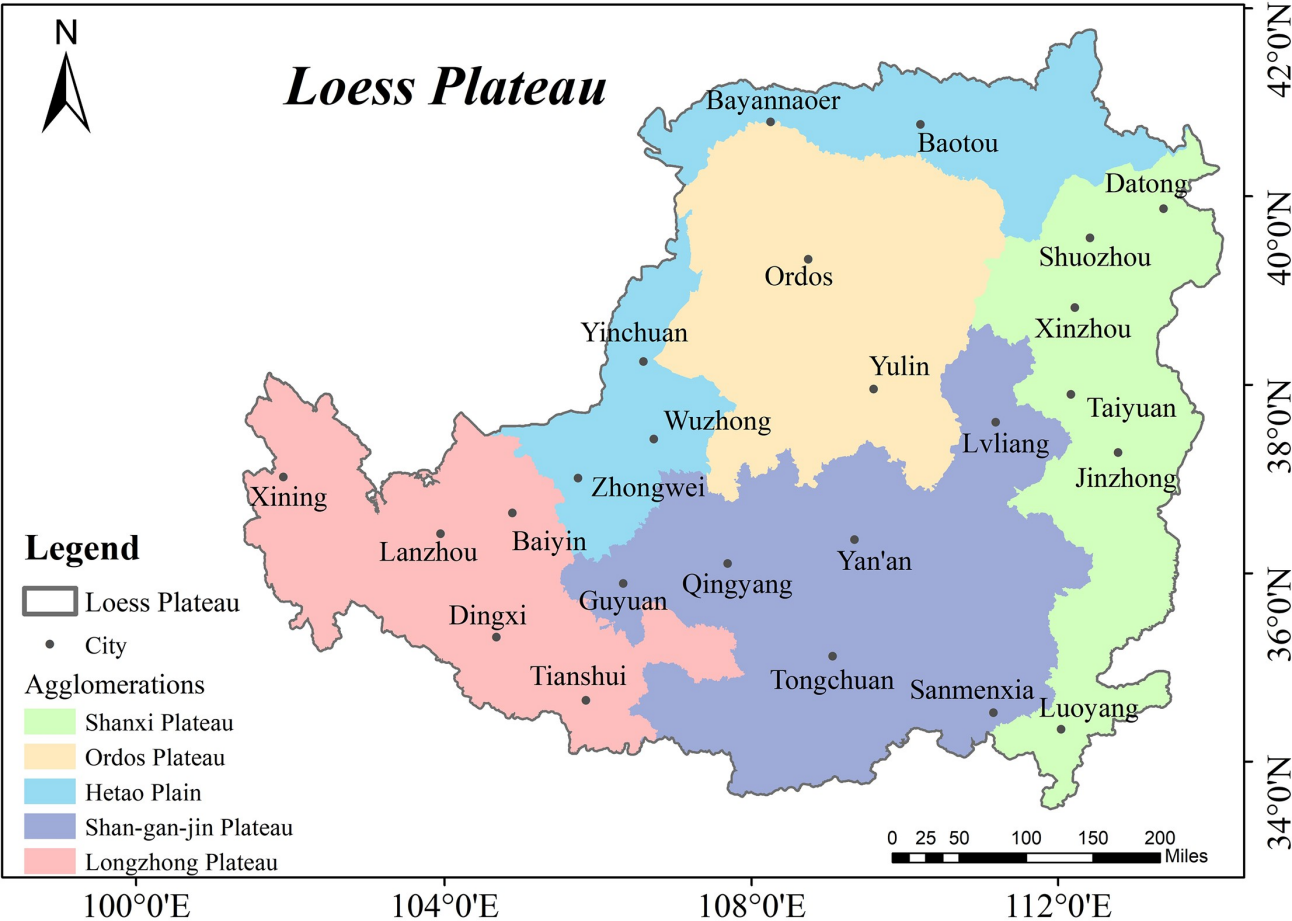
The spatial location of the Loess Plateau region and its representative cities. The base map outline was obtained by using ArcGIS 10.2 based on the Service Center of Standard Map (http://bzdt.ch.mnr.gov.cn/) and the number of the permission is GS (2020) 4621. The spatial extent of the Loess Plateau was obtained from the Resource and Environmental Science Data Center of the Chinese Academy of Sciences (http://www.resdc.cn/).

The Loess Plateau is divided into five plateau or plain areas based on regional characteristics: Shanxi Plateau, Shan-Gan-Jin Plateau, Longzhong Plateau, Ordos Plateau, and Hetao Plain. Based on the national inventory of resource-based cities (http://www.gov.cn); and taking into account the completeness, rationality, and balance of the geographical location of the five natural regions of the Loess Plateau where the evaluation is conducted; as well as the scale of the cities and the accessibility of the data, a total of 24 typical cities were selected to evaluate and analyze the RECC of the Loess Plateau. Table 1 and Fig 2 show the locations of specific natural areas and cities.

**Table 1:** Classification of natural areas in the Loess Plateau.

Natural area	Representative cities
Shanxi Plateau	Taiyuan, Datong, Shuozhou, Luoyang, Xinzhou, Jinzhong
Shan-Gan-Jin Plateau	Yan’an, Tongchuan, Qingyang, Guyuan, Sanmenxia, Lvliang
Longzhong Plateau	Lanzhou, Tianshui, Xining, Dingxi, Baiyin
Ordos Plateau	Yulin, Ordos
Hetao Plain	Bayannaoer, Baotou, Wuzhong, Zhongwei, Yinchuan

### Data sources and RECC evaluation index system construction

Most data in this paper are derived from *the China City Statistical Yearbook (2014–2019)*, *Provincial Statistical Yearbook (2014–2019)*, *Provincial Water Resources Bulletins (2013–2018)*, *and Statistical Communiques on National Economic and Social Development of Cities (2013–2018)*, and the remaining missing data are supplemented from the statistical yearbooks of each city.

The concept of comprehensive urban carrying capacity involves the relationship between urban production and life, urban construction and development, people and nature, and many other aspects [47, 48]. According to the principles of scientificity, coordination, representativeness, and operability, this study considers economy, society, resources, and the environment as the system layer. The economic carrying capacity reflects the economic strength and industrial composition of the region and is the economic foundation for other subsystems in the region. The social carrying capacity reflects the current social development of the region and people’s living standards, as well as the social pressure associated with it. According to *the Outline of the National Ecologically Fragile Area Protection Plan (2008)*, the ecological vulnerability of the ecologically fragile areas in northern China, taking the Loess Plateau as an example, is characterized by an arid climate, water shortage, loose soil structure, serious land desertification, and poor natural environment. In response to this situation, this study adopts water resources per capita, water consumption in municipal districts per unit GDP, sown area per capita, and urban construction land area per capita to measure the level of resource development in the region, while adding the index of energy consumption per unit GDP to reflect the ability of the resource system to support regional social development and the consumption of resources by the socioeconomic system. Existing studies show [49] that some cities located on the Loess Plateau are rich in natural resource reserves, and the dependence of regional economic development on non-renewable resources is serious, and the negative impact of industrial pollutant emissions on ecologically fragile areas is large, and the continuation of this development approach will lead to a vicious circle of ecology, resources, and economic development. Therefore, the ratio of industrial solid wastes comprehensively utilized, the ratio of wastewater treated at centralized sewage plants, the volume of industrial wastewater discharged per capita, the volume of SO_2_ emissions per capita, per capita area of green land, and green coverage ratio of built-up areas were used to measure the ecological development level of the study area, reflecting the degree of pollution and treatment caused to the environment in the process of socioeconomic development of the area. The specific evaluation index systems are presented in Table 2.

**Table 2:** Indicator systems of resource and environmental carrying capacity.

Target layer	System layer	Criteria layer	Basic index layer (units)	Indicator type
RECC	Economic subsystem	Economic development	Per capita GDP (yuan) (X_1_)	+
			The tertiary industry share of GDP (%) (X_2_)	+
			Expenditure for science and technology share of GDP (%) (X_3_)	+
		Social progress	Per capita general budget revenue of local finance (yuan) (X_4_)	+
			Per capita fixed asset investment (yuan) (X_5_)	+
	Social subsystem	Population pressure	Urbanization rate (%) (X_6_)	+
			Natural population growth rate (‰) (X_7_)	-
			Population density (person/km^2^) (X_8_)	-
		Health services	Number of beds of medical and health institutions (bed) (X_9_)	+
		Urban Construction	Per capita area of paved roads (m^2^) (X_10_)	+
	Resource subsystem	Water resources	Per capita water resources (m^3^) (X_11_)	+
			water consumption in municipal districts per unit GDP (ton/10,000 yuan) (X_12_)	-
		Land resources	per capita sown area (hectares/10,000 people) (X_13_)	+
			Urban construction land area per capita (km^2^/10,000 people) (X_14_)	+
		Energy resources	Energy consumption per unit GDP (a ton of standard coal/10,000 yuan) (X_15_)	-
	Environmental subsystem	Environmental governance capability	Ratio of industrial solid wastes comprehensively utilized (%) (X_16_)	+
			the ratio of wastewater treated at centralized sewage plants (%) (X_17_)	+
		Environmental pollution intensity	Volume of industrial wastewater discharged per capita (ton) (X_18_)	-
			Volume of SO_2_ emissions per capita (ton/10,000 people) (X_19_)	-
		Environmental resource supply	Per capita area of green land (hectares/10,000 people) (X_20_)	+
			Green coverage ratio of built-up areas (%) (X_21_)	+

Note: "+" represents a positive impact on RECC, "-" represents a negative impact on RECC.

### Entropy TOPSIS model

Entropy originated in physics and was later used in the multi-objective decision-making of the system. The weight is determined by collecting the raw data. The smaller the entropy index, the greater the entropy weight, which implies that the index is more important, and vice versa. The TOPSIS method is a common and effective method for multi-objective decision analysis. This study adopts the entropy-weight-based TOPSIS method, which can avoid the interference of the results caused by subjective factors, and make its conclusions objective and effective. This paper calculates the specific methods and steps for each city over the years as follows:

1. Construction of standardized evaluation matrix
  The original evaluation matrix of the RECC index is expressed as Eq (1). In the original matrix, the positive correlation index (the greater the better) (Eq (2)) and the negative correlation index (the smaller the better) (Eq (3)) are subjected to range transformation method to obtain a standardized matrix (Eq (4)).
$$U=[\begin{array}{ccc} V_{11} & \cdots & V_{1n}\\ \vdots & \ddots & \vdots \\ V_{m1} & \cdots & V_{mn} \end{array}]$$ (1)
$$r_{ij}=\frac{V_{ij}-\min(V_{ij})}{\max(V_{ij})-\min(V_{ij})}$$ (2)
$$r_{ij}=\frac{\max(V_{ij})-V_{ij}}{\max(V_{ij})-\min(V_{ij})}$$ (3)
$$R=[\begin{array}{ccc} r_{11} & \cdots & r_{1n}\\ \vdots & \ddots & \vdots \\ r_{m1} & \cdots & r_{mn} \end{array}]$$ (4)
  In Eqs (2), (3), and (4), *R* is the standardized evaluation matrix, where *r*_*ij*_ is the standardized value of the *j*_*th*_ index in the *i*_*th*_ city. *i* = 1,2,…,*m* where *m* is the number of cities evaluated, and *j* = 1,2,…,*n n* is the number of indicators evaluated. *r* represents the data values of the different evaluation indicators in different cities after the change of range.
2. Determination of indicator weights
$$W_{j}=\frac{1-e_{j}}{n-\sum_{j=1}^{n}e_{j}}$$ (5)
  In Eq (5), *W*_*j*_ is the entropy weight of the evaluation index, *e*_*j*_ is the entropy of the evaluation index, in which $e_{j}=-\frac{1}{\ln m}(\sum_{i=1}^{m}p_{ij}\ln p_{ij})$, $p_{ij}=\frac{r_{ij}}{\sum_{i=1}^{m}r_{ij}}$, when *p*_*ij*_ = 0, *p*_*ij*_ ln *p*_*ij*_ = 0.
3. Construction of the evaluation matrix
  The calculated weights are assigned to each indicator with corresponding weights through Eq (6) to objectively carry out a comprehensive evaluation.
$$Y=[\begin{array}{ccc} y_{11} & \cdots & y_{1n}\\ \vdots & \ddots & \vdots \\ y_{m1} & \cdots & y_{mn} \end{array}]=[\begin{array}{ccc} r_{11}\times W_{1} & \cdots & r_{1n}\times W_{n}\\ \vdots & \ddots & \vdots \\ r_{m1}\times W_{1} & \cdots & r_{mn}\times W_{n} \end{array}]$$ (6)
4. Determination of positive and negative ideal solutions
$$Y^{+}=\{\begin{array}{c} \max y_{ij}|i=1,2,\ldots ,m \end{array}\},1\leq i\leq m$$ (7)
$$Y^{-}=\{\begin{array}{c} \min y_{ij}|i=1,2,\ldots ,m \end{array}\},1\leq i\leq m$$ (8)
  *Y*^+^ is the maximum value of the *j*_*th*_ evaluation index in the *i*_*th*_ evaluation object (Eq (7)), and *Y*^-^ is the minimum value of the *j*_*th*_ evaluation index in the *i*_*th*_ evaluation object (Eq (8)).
5. Calculation of relative closeness
  The Euclidean metric formula (Eqs (9) and (10)) is used to calculate the distance from the evaluation vector to the positive ideal solution and the negative ideal solution for different evaluation objects, respectively, and the relative closeness (CL) is calculated by Eq (11).
$$D_{i}^{+}=\sqrt{\sum_{j=1}^{n}{\left(y_{j}^{+}-y_{ij}\right)}^{2}}$$ (9)
$$D_{i}^{-}=\sqrt{\sum_{j=1}^{n}{\left(y_{j}^{-}-y_{ij}\right)}^{2}}$$ (10)
$$CL_{i}=\frac{D_{i}^{-}}{D_{i}^{+}+D_{i}^{-}}(0\leq CL_{i}\leq 1,i=1,2,\ldots ,m)$$ (11)
  CL is the degree of the evaluation object close to the optimal ideal solution, and its value ranges from 0 to 1. In Eq (11), the closer the CL is to 1, the closer the RECC of the city is to the optimal carrying capacity level, and the closer the CL is to 0, the closer the RECC of the city is to the worst carrying capacity level. The RECC can be judged and ranked as superior or inferior according to the size of the CL of each city.

### Obstacle factors diagnosis model

Based on the analysis of the temporal and spatial changes of RECC over the years, to further explore the obstacles restricting the improvement of RECC in the Loess Plateau, the obstacle degree model is introduced to diagnose the obstacle factors [50, 51]. The obstacle degree model is expressed as in Eq (12).


$$P_{jk}=\frac{(1-r_{jk})\times w_{j}\times 100\%}{\sum_{j=1}^{n}\left(1-r_{jk}\right)\times w_{j}}$$ (12)

Where, *w*_*j*_ is the weight of the *j*_*th*_ indicator, and *p*_*jk*_ is the obstacle degree of the *j*_*th*_ index in the *k*_*th*_ year.

## Results

### RECC of 24 cities

**Fig 3:**
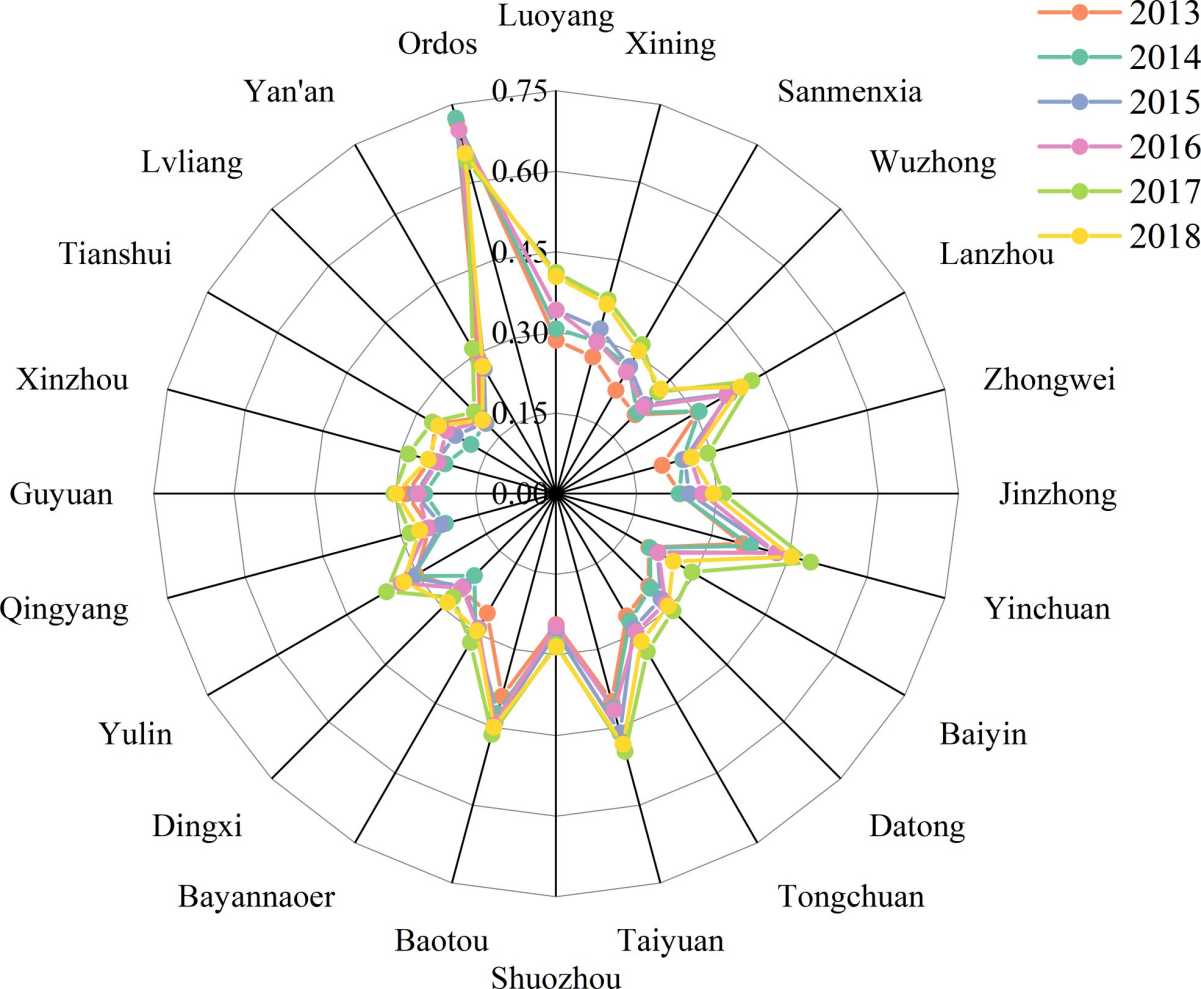
The calculation results of resource and environmental carrying capacity in 24 cities from 2013 to 2018. Note: The growth rate of the carrying capacity of each city decreases clockwise at 12 o’clock. Same as below.

The carrying capacity index of each subsystem and the RECC index of the 24 cities were calculated using the abovementioned model. In general, the RECC of most cities are on the rise (Fig 3, Table 3). The average RECC index for the 24 cities increased from 0.2860 in 2013 to 0.3306 in 2018, indicating an increase of 15.59% over six years, with the overall regional RECC still at a low level. The carrying capacity of various subsystems and the RECC of Ordos ranked first during the six years, and their performance is relatively excellent. Taiyuan, Baotou, and Yinchuan ranked second, third, and fourth, respectively. Among all cities, Luoyang has the highest growth rate of index value over six years. The largest increase in the ranking is in the case of Sanmenxia, which rose from 20th in 2013 to 10th position in 2018. In addition, there is a significant difference in RECC among the 24 cities. In 2018, for example, Ordos (0.6554), and Lvliang (0.1944) have the best and the worst carrying capacities, respectively. The problem of uneven regional development in the Loess Plateau is evident.

**Table 3:** The growth rate of the carrying capacity of each subsystem in 24 cities from 2013 to 2018 (%).

	Economic subsystem	Social subsystem	Resource subsystem	Environmental subsystem	RECC
Luoyang	73.21	48.52	10.62	26.40	41.53
Xining	63.54	41.18	15.73	2.04	38.53
Sanmenxia	55.37	26.98	-5.21	47.97	38.01
Wuzhong	77.06	33.56	-3.80	-0.52	32.54
Lanzhou	52.19	28.77	10.20	20.19	30.20
Jinzhong	81.38	22.46	-5.79	-0.35	27.80
Yinchuan	30.71	43.83	-24.26	27.71	26.58
Zhongwei	29.58	32.02	10.35	1.02	26.23
Baiyin	26.57	48.83	10.12	9.73	26.18
Datong	48.44	37.12	-11.77	-5.24	21.36
Tongchuan	125.94	37.94	-8.44	-11.07	20.92
Taiyuan	24.95	28.17	4.42	2.44	20.63
Shuozhou	16.95	61.64	-5.23	-15.60	17.06
Baotou	3.69	28.52	-2.33	10.54	15.19
Dingxi	-14.10	31.26	12.61	-14.33	15.00
Bayannaoer	-5.77	58.88	-10.98	16.34	14.64
Qingyang	21.90	43.19	-25.69	-50.11	8.47
Yulin	-42.14	83.41	-17.76	8.02	7.83
Guyuan	-8.29	134.27	-24.72	4.41	6.62
Xinzhou	-16.87	52.02	-29.23	0.58	1.95
Tianshui	2.87	70.70	-40.09	-7.01	-2.30
Lvliang	-12.74	53.64	-31.56	-21.79	-3.20
Yan’an	4.22	48.00	-40.93	-9.05	-5.96
Ordos	-1.70	-16.18	-8.12	-9.84	-8.65

**Fig 4:**
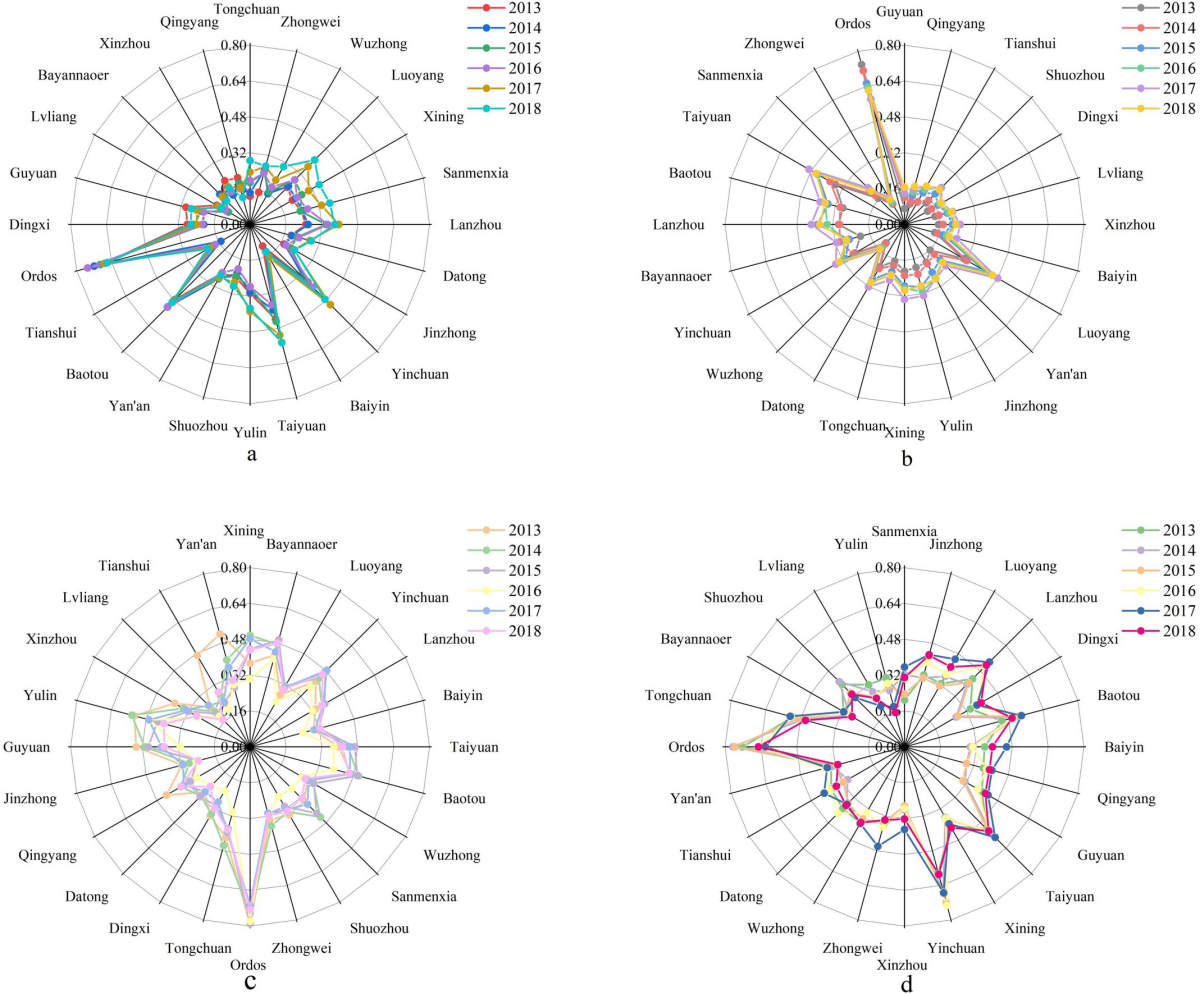
The calculation results of each subsystem carrying capacity in 24 cities from 2013 to 2018. (a) Economic carrying capacity; (b) Social carrying capacity; (c) Resource carrying capacity; (d) Environmental carrying capacity.

From the perspective of economic subsystem, the carrying capacity of the economic subsystem in most cities (70.83%) has increased to varying degrees over six years (Fig 4, Table 3), among which Tongchuan has the highest economic subsystem index growth rate among the 24 cities. In 2018, the average carrying capacity of all urban economic subsystems increased by 20.85% compared to 2013. Tongchuan and Datong are the only cities where the carrying capacity of the economic subsystem has been increasing continuously over the past six years.

From the perspective of social subsystem, the urban agglomerations showed a rapid and positive development trend from 2013 to 2018 (Fig 4, Table 3). In 2018, the average carrying capacity of all urban social subsystems increased by 33.58% compared to 2013, but was still low (0.2752). After 2013, the carrying capacity of the Ordos social subsystem showed a continuous downward trend, and it rebounded slightly in 2018. The carrying capacity of the social subsystem of the urban agglomeration tended to be average. The city with the lowest social subsystem carrying capacity in 2013, Guyuan had the highest growth rate of the social subsystem carrying capacity and moved up two places in the ranking.

From the perspective of resource and environmental subsystems (Fig 4, Table 3), 70.83% of the cities have a downward trend in the resource subsystem carrying capacity, with the highest growth rate of Xining, the capital of Qinghai Province, followed by Bayannaoer and Luoyang. According to the 2018 data, except for Ordos, the carrying capacity of the resource subsystems in Bayannaoer, Yinchuan, Baotou, Xining, and Taiyuan are relatively high (all above 0.4). Moreover, 29.17% of the urban resource subsystem carrying capacity index values are below 0.3, which is a relatively low level. Some cities in the upper reaches of the economic ranking, such as Ordos and Yulin, have experienced a decline in the carrying capacity of the environmental subsystem. Meanwhile, some cities, such as Bayannaoer, experienced a decline in the carrying capacity of the environmental subsystem when the carrying capacity of the resource subsystem increased. Statistics show that the carrying capacity of the environmental subsystem in Sanmenxia has the highest growth rate in six years, while Yulin has the largest decline. The average carrying capacity of all urban environmental subsystems was virtually unchanged in 2018 compared to 2013 (an increase of 0.36%).

### RECC analysis of five natural areas

**Fig 5:**
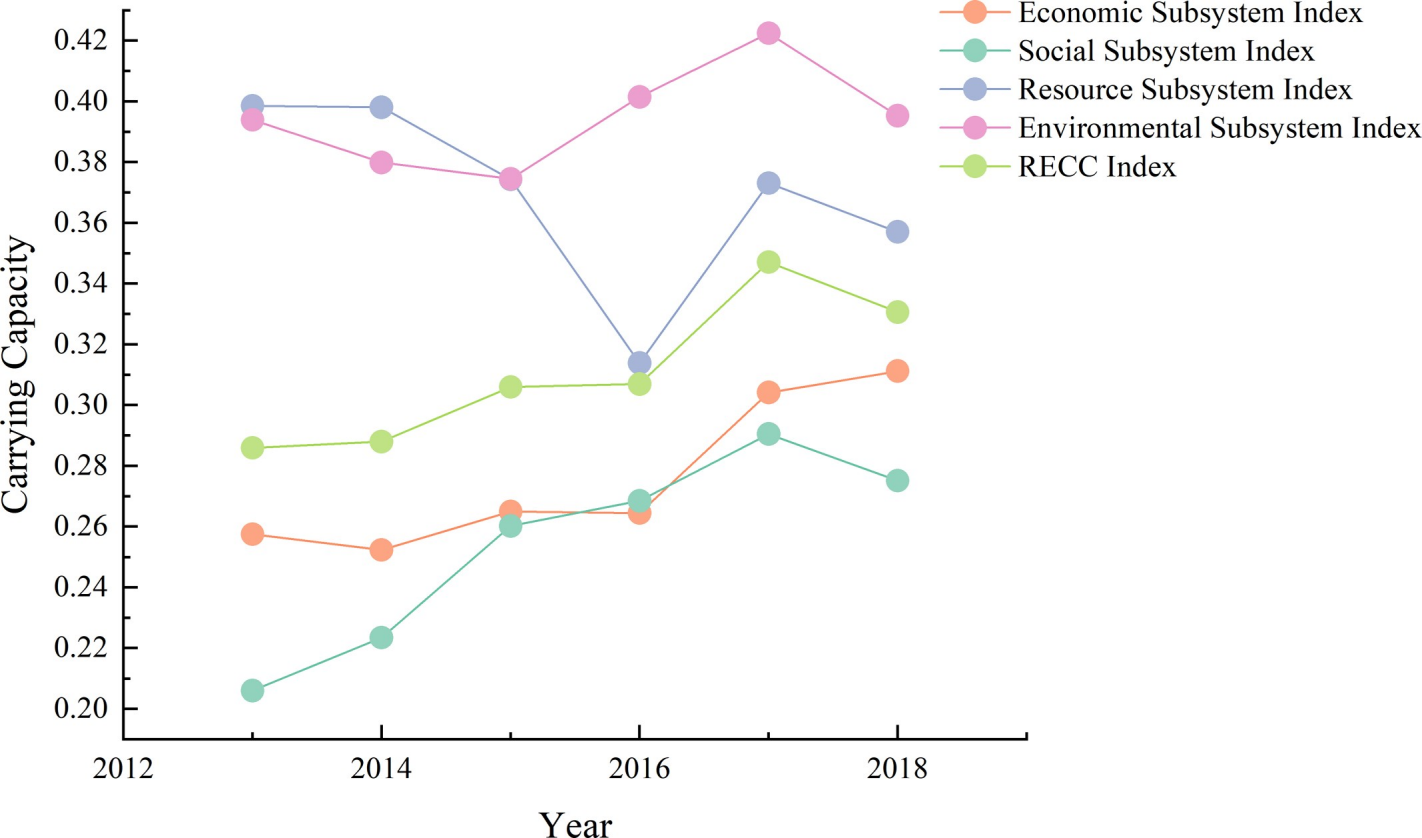
Average resource and environmental carrying capacity in 24 cities from 2013 to 2018.

From 2013 to 2018, the carrying capacity of the economic and social subsystems of the Loess Plateau region rose, the carrying capacity of the resource subsystems dipped, and the carrying capacity of the environmental subsystems remained generally stable (Fig 5). The RECC increased by 15.61% over six years. Overall, the carrying capacity of the resource and environmental subsystems exceeded the average RECC level. From the perspective of growth rate, from 2013 to 2018, the carrying capacity of each subsystem is in the descending order of social, economic, environmental, and resource subsystems.

**Fig 6:**
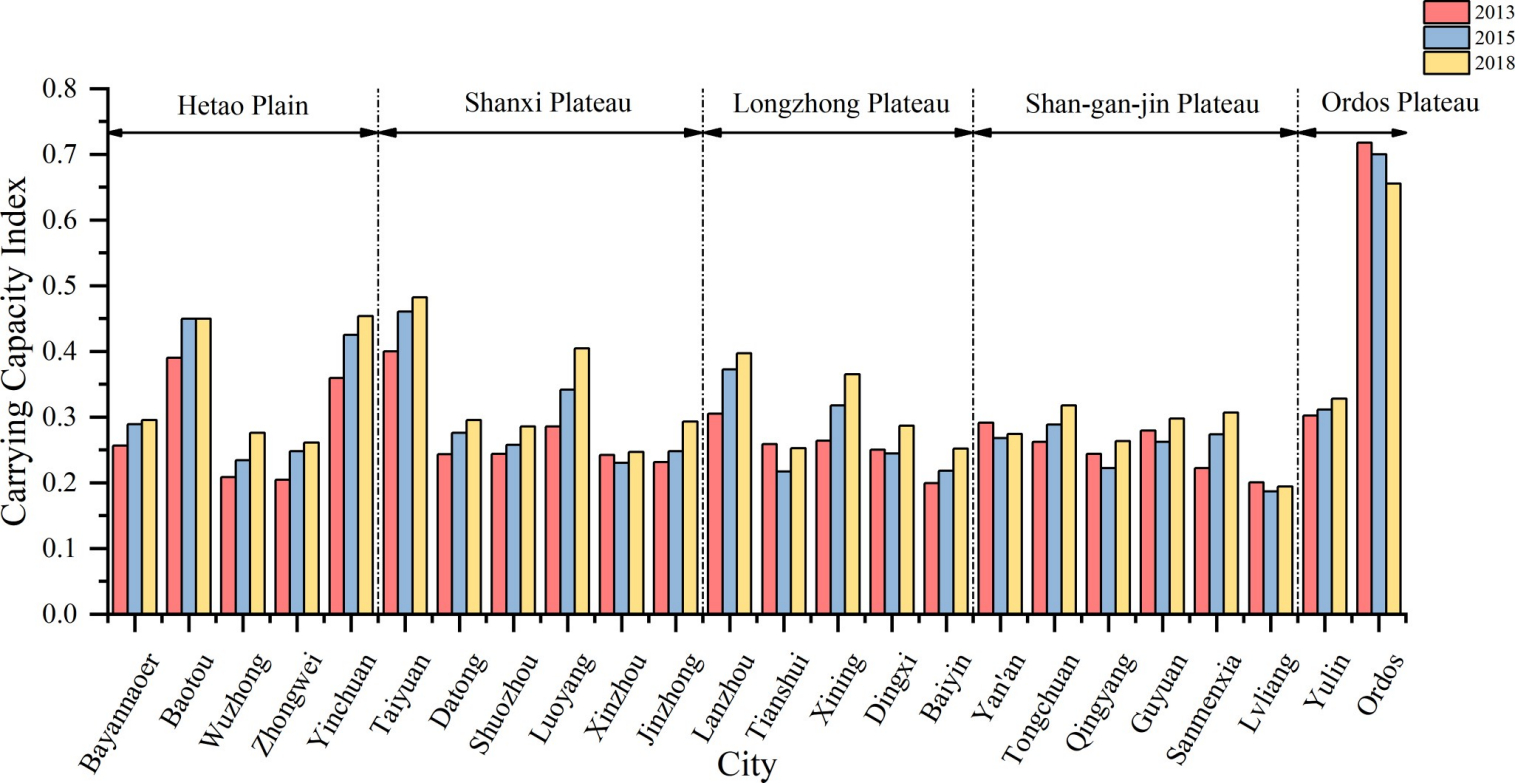
Performances of the resource and environmental carrying capacity of 24 cities in 2013, 2015, and 2018.

**Fig 7:**
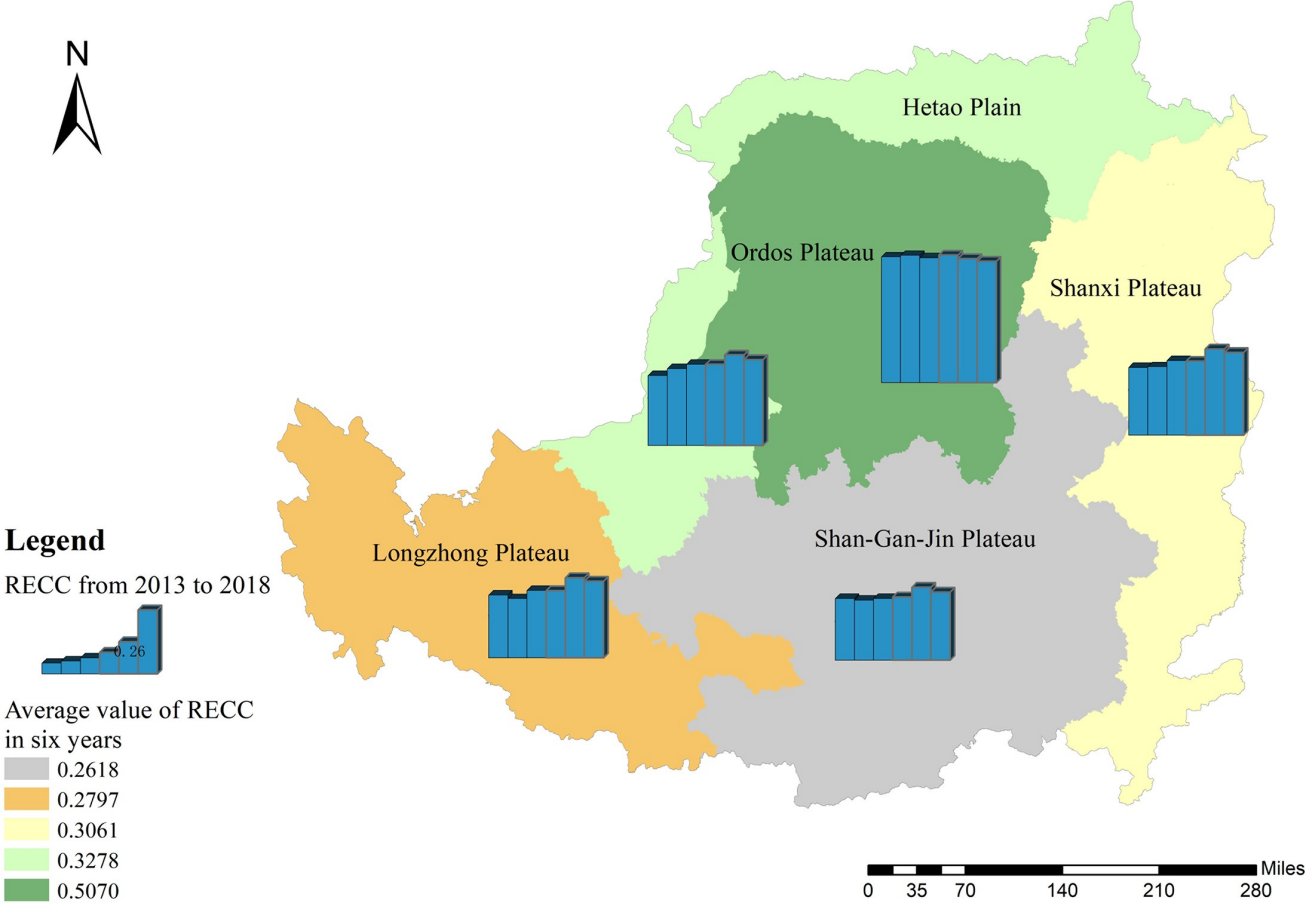
Temporal and spatial pattern of resource and environmental carrying capacity index. The image was obtained by using ArcGIS 10.2 through the open-access data process. The spatial extent of the Loess Plateau was obtained from the Resource and Environmental Science Data Center of the Chinese Academy of Sciences (http://www.resdc.cn/).

From 2013 to 2018, Hetao Plain, Shanxi Plateau, Longzhong Plateau, and Shan-Gan-Jin Plateau showed an upward RECC trend, and their overall growth rates decreased sequentially (Figs 6 and 7). The Ordos Plateau remained stable with a decrease of 3.57% over six years. The data show that from 2013 to 2018, the average RECC index of the Ordos Plateau was the highest (0.5070), and the Shan-Gan-Jin Plateau was the lowest (0.2618).

#### Economic subsystem

**Fig 8:**
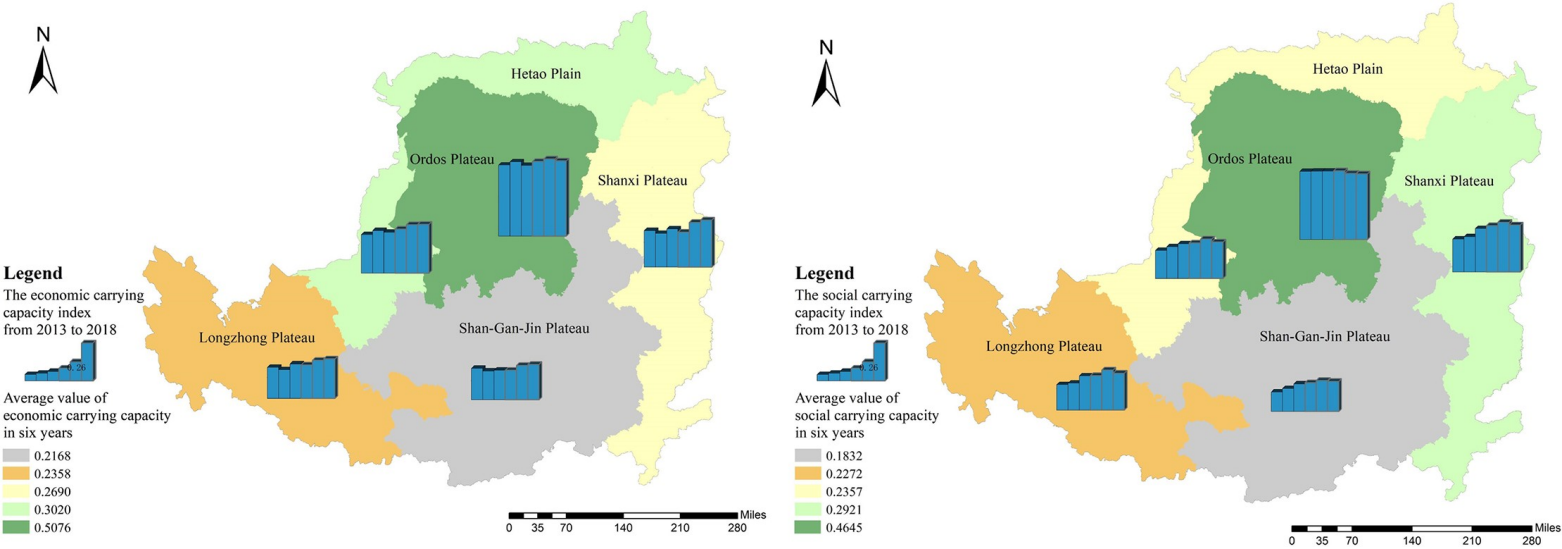
Temporal and spatial pattern of carrying capacity (left: economic carrying capacity; right: social carrying capacity). The image was obtained by using ArcGIS 10.2 through the open-access data process. The spatial extent of the Loess Plateau was obtained from the Resource and Environmental Science Data Center of the Chinese Academy of Sciences (http://www.resdc.cn/).

The carrying capacity of the economic subsystems of the five natural areas showed an overall upward trend from 2013 to 2018 (Fig 8). The Shanxi Plateau had the highest overall growth rate of 28.56%. The data show that the economic subsystem carrying capacity index of the Ordos Plateau is the highest, reaching 0.5167 in 2018, which is much higher than the other four natural areas.

#### Social subsystem

The carrying capacity of the social subsystems of the five natural areas showed an overall upward trend from 2013 to 2018 (Fig 8). The Shan-Gan-Jin Plateau had the highest overall growth rate of 54.37%. The overall carrying capacity of the social subsystem of the Ordos Plateau has not changed much in six years. It increased slightly from 2014 to 2016 and then showed a downward trend over the next two years. The Ordos Plateau has the highest social subsystem carrying capacity, with an average value of 0.4645 over six years. The growth rate of the social subsystems of Guyuan and Qingyang in the Shan-Gan-Jin Plateau ranks the highest among the 24 cities, increasing the index value of the Shan-Gan-Jin Plateau substantially.

#### Resource subsystem

**Fig 9:**
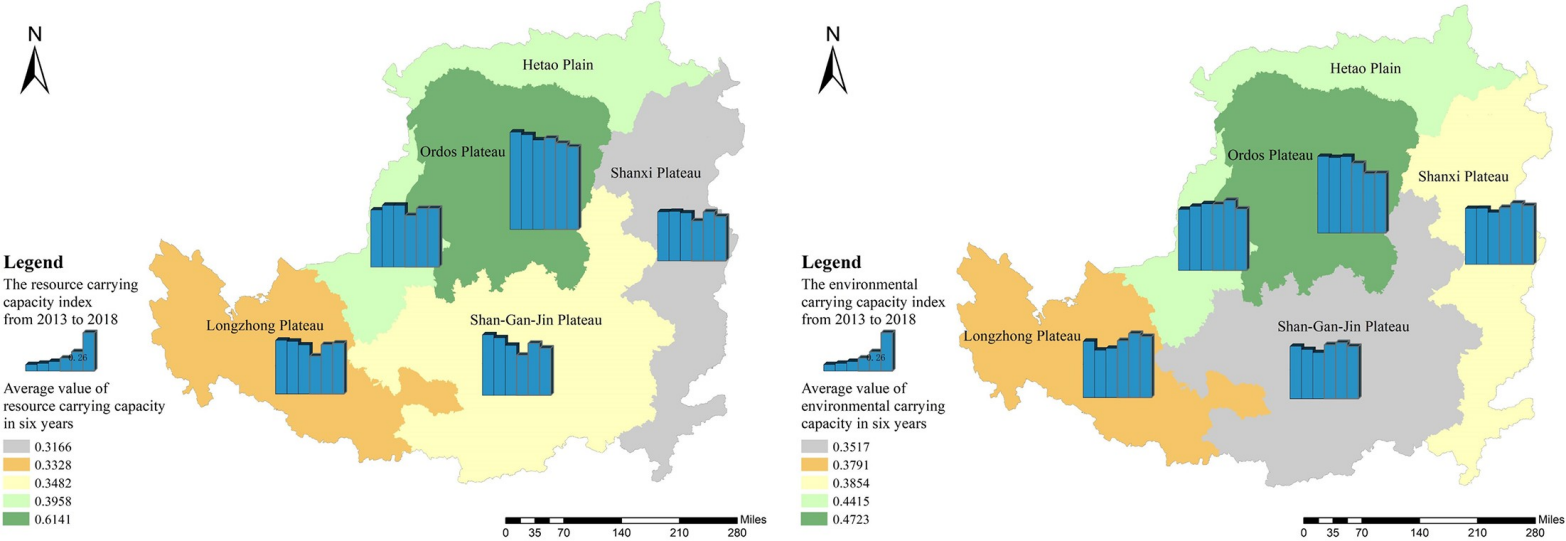
Temporal and spatial pattern of carrying capacity (left: resource carrying capacity; right: environmental carrying capacity). The image was obtained by using ArcGIS 10.2 through the open-access data process. The spatial extent of the Loess Plateau was obtained from the Resource and Environmental Science Data Center of the Chinese Academy of Sciences (http://www.resdc.cn/).

From 2013 to 2018, the resource subsystem carrying capacity of only the Hetao Plain increased slightly, while the index value of the other natural areas decreased to varying degrees. Fig 9 shows that the overall decline rate of the Shan-Gan-Jin Plateau is the highest at 22.15%. The resource subsystem carrying capacity index of the Ordos Plateau is the highest, with an average value of 0.6414 in six years, which is far ahead of the other areas.

#### Environmental subsystem

From 2013 to 2018, the environmental subsystems carrying capacity of the Longzhong Plateau and Shanxi Plateau showed an upward trend, while that of the Hetao Plain and the Shan-Gan-Jin Plateau remained stable, and that of the Ordos Plateau showed a downward trend (Fig 9). Overall, the difference in the carrying capacity of environmental subsystems among the five natural areas is relatively small, which is the index with the smallest difference among all the subsystems. In the six years, the highest average value of the environmental subsystem index is in the case of Ordos Plateau (0.4723), and the Shan-Gan-Jin Plateau has the lowest average value (0.3517). From the perspective of resource and environmental systems, the best performing area is the Hetao Plain, where the carrying capacities of the resource and environmental subsystems have both improved. In areas where the carrying capacity of the environmental subsystem increased (Longzhong Plateau and Shanxi Plateau), the carrying capacity of the resource subsystem showed a downward trend. The carrying capacity of the resource and environmental subsystems of the Shan-Gan-Jin Plateau and the Ordos Plateau also decreased. This shows that the economic-resource-environmental development in most areas of the Loess Plateau is not coordinated.

### Obstacle factors diagnosis

According to the obstacle factors diagnosis model, we measured the RECC index data of 24 cities from 2013 to 2018 to explore and identify obstacles restricting the improvement of RECC in these cities, which could put forward reasonable countermeasures and suggestions for the optimal development of the Loess Plateau.

**Fig 10:**
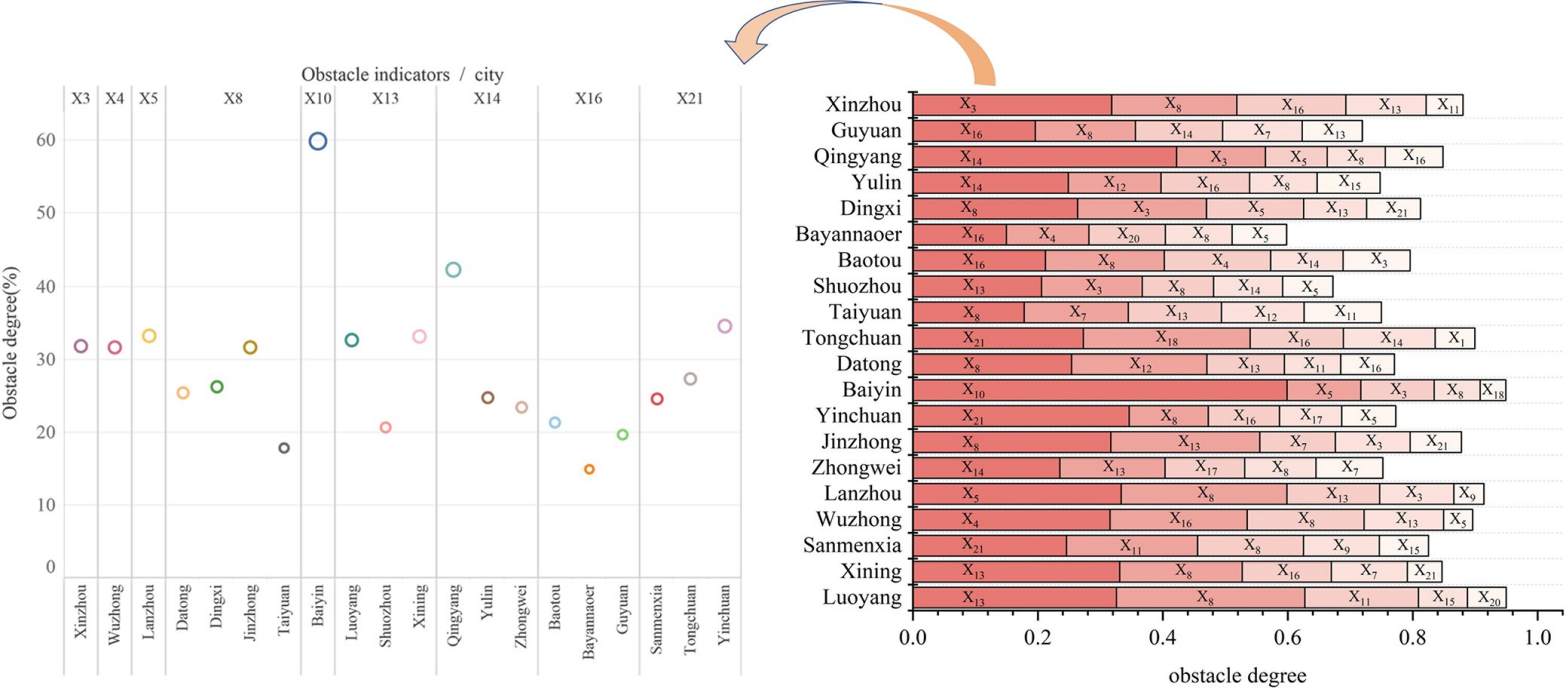
Obstacle indicators for resource and environmental carrying capacity in 20 cities in 2018 (top five).

Twenty cities demonstrated improved RECC from 2013 to 2018. Fig 10 and S1 Table shows the top five obstacles for these cities. The primary obstacles belong to resource subsystem (in six cities), environmental subsystem (in six cities), and social subsystem (in five cities). Resources, environmental, and social factors put pressure on the improvement of RECC in most cities.

The left side of Fig 10 shows the primary obstacle indicators for each city. The top obstacle factor is population density (X_8_), which is the primary obstacle to the improvement of RECC in Jinzhong, Datong, Taiyuan, and Dingxi. The four indicators of per capita sown area (X_13_), per capita urban construction land area (X_14_), ratio of industrial solid wastes comprehensively utilized (X_16_), and green coverage ratio of built-up areas (X_21_), all appear three times. The largest value among all obstacle indicators is the per capita area of paved roads in Baiyin (X_10_), which reached 59.88%.

Tianshui, Lvliang, Yan’an, and Ordos showed a downward trend in the RECC from 2013 to 2018. As shown in Table 4, the main obstacles affecting these four cities in recent years are resource, environmental, and social factors. Tianshui and Ordos have the most indicators belonging to the resource subsystem, accounting for 36.67% in both cases, while Lvliang and Yan’an have the most indicators belonging to the environmental subsystem, accounting for 33.33% and 36.67%, respectively. The obstacle factors belonging to the social subsystem rank second in Tianshui, Yan’an, and Ordos, and account for 30%, 23.33%, and 30%, respectively.

**Table 4:** Obstacle indicators for cities with negative resource and environmental carrying capacity growth from 2013 to 2018 (top five).

Year			2013	2014	2015	2016	2017	2018
items			obstacle indicators / obstacle degree					
Obstacle ranking	Tianshui	1	X_7_/12.20	X_7_/11.50	X_7_/14.94	X_11_/17.63	X_11_/17.16	X_3_/25.07
		2	X_18_/9.34	X_18_/8.47	X_11_/11.27	X_7_/16.24	X_7_/15.53	X_8_/16.01
		3	X_14_/7.91	X_11_/8.07	X_18_/11.18	X_3_/12.95	X_3_/12.20	X_13_/14.35
		4	X_2_/7.26	X_14_/7.95	X_1_/9.46	X_9_/9.20	X_14_/9.35	X_16_/13.86
		5	X_17_/6.58	X_9_/7.16	X_14_/8.16	X_14_/7.30	X_8_/8.88	X_11_/12.78
	Lvliang	1	X_20_/14.83	X_20_/12.98	X_20_/11.21	X_20_/13.40	X_8_/17.44	X_13_/24.02
		2	X_14_/10.52	X_14_/8.83	X_21_/8.16	X_21_/9.61	X_7_/15.18	X_8_/23.00
		3	X_19_/9.18	X_11_/8.75	X_11_/8.02	X_4_/8.63	X_13_/14.66	X_3_/20.04
		4	X_9_/9.02	X_19_/7.98	X_4_/7.19	X_14_/8.63	X_16_/10.59	X_11_/8.67
		5	X_10_/7.75	X_9_/7.53	X_1_/6.79	X_1_/7.39	X_4_/8.49	X_16_/7.44
	Yan’an	1	X_12_/13.41	X_12_/17.21	X_12_/14.88	X_12_/15.90	X_5_/15.61	X_17_/19.78
		2	X_5_/9.98	X_20_/10.76	X_17_/13.22	X_17_/12.17	X_10_/12.52	X_8_/15.63
		3	X_20_/8.23	X_21_/9.73	X_10_/8.89	X_5_/10.22	X_8_/11.99	X_3_/14.09
		4	X_21_/7.67	X_13_/7.05	X_19_/7.52	X_10_/8.24	X_17_/11.61	X_5_/13.93
		5	X_14_/6.55	X_10_/6.09	X_20_/6.96	X_8_/7.31	X_3_/6.74	X_16_/10.26
	Ordos	1	X_11_/13.11	X_14_/19.19	X_11_/18.04	X_14_/25.13	X_14_/16.87	X_14_/29.32
		2	X_19_/11.77	X_11_/14.75	X_19_/12.73	X_18_/12.45	X_11_/11.84	X_8_/14.17
		3	X_7_/10.26	X_19_/10.58	X_13_/8.67	X_3_/10.38	X_7_/10.30	X_18_/9.78
		4	X_13_/9.74	X_7_/8.82	X_5_/7.85	X_7_/9.82	X_20_/8.24	X_10_/9.35
		5	X_20_/9.16	X_13_/8.57	X_9_/6.26	X_8_/8.01	X_8_/7.15	X_1_/8.97

In 2018, the five major obstacles to the improvement of RECC in the four cities are expenditures for science and technology share of GDP (X_3_), population density (X_8_), per capita sown area (X_13_), ratio of industrial solid wastes comprehensively utilized (X_16_), and per capita water resources (X_11_), among which the indicator of population density (X_8_) appears in all four cities. This indicates that the Loess Plateau is facing a severe population concentration problem, which has become the primary factor restricting the improvement of RECC in the region.

## Discussion

### Specific issues in the social context

The results show that the carrying capacity of the economic subsystem of the Ordos Plateau is significantly higher than that of other natural areas. According to the latest data from the comprehensive statistical information of cities in western China, in 2019, Yulin and Ordos, located in the Ordos Plateau, ranked sixth and eighth among the cities in western China with a GDP of 413.628 billion Yuan and 360.503 billion Yuan, respectively. In the Loess Plateau, Ordos lies second only to Xi’an, and its economy is developing well. The resources and energy advantages of the Ordos Plateau have made significant contributions to the economic development of the region. The outstanding performance of the economic subsystem carrying capacity of the Hetao Plain is due to its developed economy, with fertile soil, developed animal husbandry and aquaculture; and it is also the most important agricultural area in Northwest China. The carrying capacity of the economic subsystem of Tongchuan City, which is located on the Shan-Gan-Jin Plateau, has increased the most. This is mainly due to the implementation of the Western Development Strategy, the economic construction goals set out in the 13th Five-Year Plan for National Economic and Social Development, and Tongchuan City’s support for high-tech industries in recent years.

From the perspective of social subsystems, Guyuan performed the best in recent years. The reason is that between 2013 and 2018, the per capita area of paved roads and the number of beds in medical and health institutions in Guyuan city increased significantly, the allocation of health resources was more reasonable, and the level of urban construction continued to increase. The urbanization rate of Guyuan has increased by 44.72% in six years, meeting the social development and increased service demand of the people. For Qingyang, which ranks second in the growth rate of the social subsystem carrying capacity, its excellent performance is due to a 36.87% decrease in the natural population growth rate over six years, while the urbanization rate increased by 29.77%. The number of beds in medical and health institutions has increased by 53.23% in six years, and people’s living conditions have improved.

From the perspective of resource subsystem, the Shan-Gan-Jin Plateau declined the most. This is because the plateau has serious soil erosion, low vegetation coverage, and the destruction of forestry resources, which restrict the carrying capacity of its resource subsystem. The total water resources per capita of Yan’an and Sanmenxia, located in the Shan-Gan-Jin Plateau, have different degrees of decline, while Lvliang, Guyuan, and Qingyang are limited by the impact of the reduction in the sown area per capita. Yan’an is the city with the largest decrease in the carrying capacity of the resource subsystem among all cities, with its total water resources per capita decreasing by nearly half (47.28%) over the six years, and the water consumption per unit of GDP in the municipal area decreasing by 36.87% over the same period. The scarcity of water resources is the main factor inhibiting the increase in the carrying capacity of the resource subsystem in Yan’an.

From the perspective of environmental subsystems, the Ordos Plateau declined the most. This is because the carrying capacities of the environmental subsystem of Ordos and Yulin in the Ordos Plateau have different degrees of decline. In addition, the ecological environment of the loess area is very fragile. At the same time, ecological environmental protection measures during resource extraction are not sufficient, which causes the ecological environment to deteriorate, and further leads to a decline in the carrying capacity of the environmental subsystem. Yulin is the city with the highest decline among the 24 cities. The comprehensive utilization rate of general industrial solid waste in Yulin City continued to decline in 2017 and 2018, and its utilization rate in 2018 was only 30.29% in 2013. According to the *Bulletin of the State of the Ecological Environment in Shaanxi Province in 2018*, the comprehensive index of environmental air quality in Yulin City in 2018 was 5.38 lower than the average value of 5.72 in Shaanxi Province. The number of days with good ambient air quality in Yulin City decreased by 13 days in 2018 compared to 2017, the largest decrease in the cities of Shaanxi Province. Yulin’s ecological environment index also ranks low in the province. In general, the carrying capacity of the environmental subsystem of the Ordos Plateau needs to be further strengthened.

### Conceptual framework of the RECC influence mechanism

The RECC system is divided into two parts: the carried subject and carried object. The carried subject includes the resource and environmental system, reflecting their ability to support the RECC system, while the carried object includes economic and social systems, reflecting their bearing pressure on the RECC system. This study used the entropy-weight-based TOPSIS model to measure and analyze the urban RECC in the Loess Plateau and found that Ordos is the city with the best RECC. Although its RECC has declined in the past two years, its index has remained almost stable from 2013 to 2016, showing an inverted "U" shape, which is similar to existing research [52]. The carrying capacity of the resource subsystem has shown a decreasing trend in recent years. According to indicator data, Datong, Shuozhou, Yan’an, and Tianshui face problems such as reduction of total water resources. Taiyuan, Lvliang, Jinzhong, Xinzhou, Baotou, and Dingxi face problems such as reduction in sown area, which is a common problem faced by most first-tier cities in China, akin to existing research [53, 54]. In addition, this paper introduces the obstacle factors diagnosis model and finds that population density (X8) is the main obstacle to the improvement of RECC for most cities. Existing studies have shown that the decline in population density in urban built-up areas over time is a global phenomenon and a long-term historical trend [55]. China is undergoing a rapid urbanization phase; with an increase in population and rapid concentration of population in cities; therefore, urban built-up areas will inevitably be affected under a certain administrative boundary, making the conflict between population and environment increasingly prominent [56]. The Loess Plateau is restricted by resources, especially arable land resources, and has limited space for urban land expansion. In the process of urban growth, the phenomenon of decreasing population density in urban built-up areas over time will intensify urban sprawl, which in turn will put enormous pressure on the arable land resources and ecological environment in and around the cities, and seriously hinder the sustainable development of the region. For cities with a low (Xinzhou) or negative (Tianshui, Lvliang, Yan’an) RECC growth rate, the investment in technological innovation (X3) also plays a major inhibitory role. Socioeconomic development enhances a contradiction between the continuous growth of resource consumption and domestic supply. To continuously improve the level of RECC and resource management, innovation-driven development and increased investment in science and technology need urgent implementation. The difference between this study and the existing research [57] is that RECC fluctuations in Qingyang, Dingxi, and Tianshui are more frequent as a result of the different research areas and research time. In addition, this study found that cities with better RECC on the Loess Plateau are primarily located in the north, such as Ordos, Baotou, Taiyuan, and Yinchuan, whereas cities with higher RECC growth rates in recent years, such as Luoyang, Xining, Sanmenxia, Lanzhou, and Zhongwei, are mainly located in the south. Most of the cities located in the northern part of the Loess Plateau are rich in resources. Ordos, with rich energy resources and many pillar industries such as coal, chemicals, and natural gas, along with Baotou and Taiyuan, which have well-developed heavy industry and energy metallurgy industries, has a relatively excellent level of individual carrying capacity. The waters of the Yellow River, which travel northwards, have irrigated a great deal of land in the Hetao Plain and nurture Yinchuan, an important city on the Loess Plateau in the northwest. In recent years, cities located in the south have issued numerous policy advices to promote the efficient use of resources and the green transformation of the economy. Luoyang, which ranks first in the RECC growth rate, has increased the proportion of tertiary industry to GDP by 46.48% and Xining by 37.30% in six years, while the two cities with the best RECC, Ordos and Taiyuan, have only increased this figure by 18.36% and 12.96%, respectively. Therefore, optimizing and adjusting the industrial structure, increasing technological innovation, developing low-carbon industries, increasing the proportion of tertiary industries, easing the pressure on resources and the environment, and at the same time strengthening the combination of ecological and environmental management, and economic development to avoid duplication of construction and waste of resources are important ways to improve RECC.

**Fig 11:**
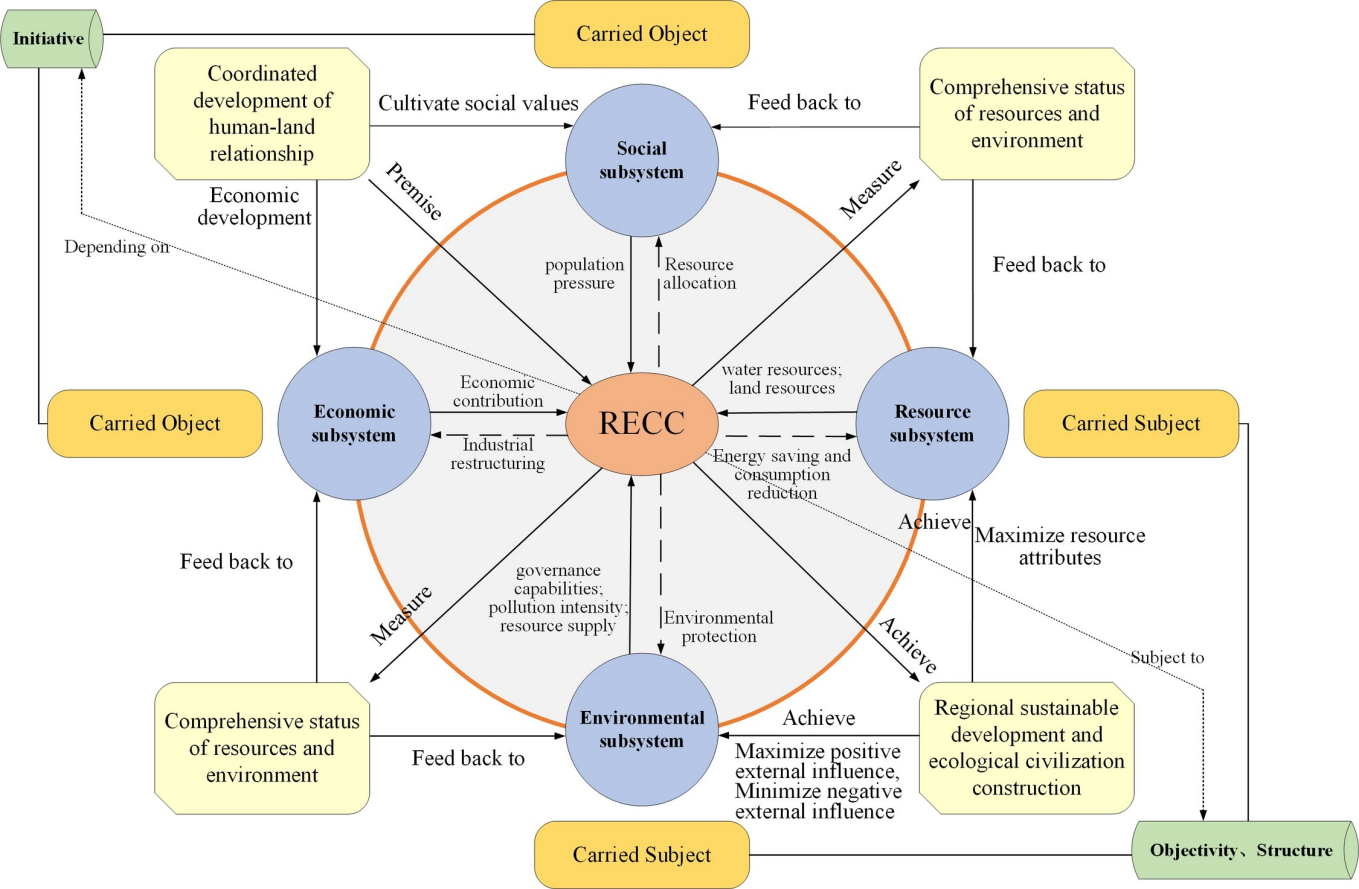
Conceptual framework for resource and environmental carrying capacity impact mechanism.

The coordinated development of human-land relationships has a positive effect on sustainable economic development and the cultivation of social values. It is also a prerequisite for improving regional RECC, and is a guarantee for regional sustainable development and ecological civilization construction. The construction of ecological civilization must take into account economic and social development, and the protection of resources and the environment; respect the rigid constraints of resources and the environment; and correctly understand the regional RECC (Fig 11). Based on the empirical analysis of the Loess Plateau, this study finds that the pressure from the social subsystem and the constraints of the resource and environmental subsystems mainly restrict the improvement of RECC. Among them, population pressure, water resources, land resources, environmental governance capabilities, environmental pollution intensity, and environmental resource supply are particularly prominent. The results show that the current economic-resource-environmental development of the Loess Plateau is not coordinated, and the problem between resource exploitation and environmental protection is a prominent contradiction. The coordinated development and synergy among regional economic and social factors, resource factors, and ecological environment factors are key to achieving sustainable development.

### Limitations

This study establishes a specific index system to evaluate the regions and cities of the Loess Plateau to analyze and compare the actual RECC levels in various regions more comprehensively. However, it should be noted that this study selected cities as representative objects and did not take into account the vast rural areas. The RECC evaluation of the entire Loess Plateau needs to be strengthened further, and the consideration of rural area indicators can be added in the future. In addition, this study did not choose cities such as Xi’an, Xianyang, Baoji, and Weinan located in the Loess Plateau to participate in the evaluation because they are geographically closer to the Guanzhong Plain and are representative cities in the Guanzhong Plain urban agglomeration, which can be included in the comprehensive analysis when discussing the carrying capacity of the Loess Plateau in the future.

## Conclusions and policy implications

### Main conclusions

This study selected 24 representative cities in the five natural areas of the Loess Plateau, established an index system that includes economic, social, resource, and environmental carrying capacity, and used the entropy-weight-based TOPSIS model and obstacle factors diagnosis model to evaluate the RECC and diagnose its inhibited factors that could support regional sustainable development. The main conclusions of this study are as follows.

1. Ordos is the city with the best RECC on the Loess Plateau. However, the carrying capacity of each subsystem and the RECC have been decreasing.
2. In the Loess Plateau, except for the Ordos Plateau, the RECC of all other natural areas have increased. The highest growth rate is 22.32% in Hetao Plain, and the least growth rate is 10.22% in Shan-Gan-Jin Plateau.
3. The contradiction between economic development and the environment in the Loess Plateau is evident. The overall RECC of the Loess Plateau is relatively low. The average RECC index in 2018 was 0.3306, with an increase of 15.61% in six years. The rate of increase is low, and there is still a lot of room for improvement.
4. Population density, investment in technological innovation, per capita sown area, and per capita water resources are the main obstacles to the improvement of RECC.

### Policy implications

The research results show that regional economic and social development levels, resource optimization capabilities, and environmental pollution control efforts will affect regional RECC to varying degrees. To improve RECC continuously, this study puts forward the following suggestions.

First, the focus should be on solving the contradiction between resources and the environment in the Loess Plateau. For Bayannaoer, Baotou, Sanmenxia, Dingxi, Jinzhong, and Guyuan, resource subsystem carrying capacity and environmental subsystem carrying capacity did not increase simultaneously. In the future, the market should play a decisive role in the allocation of planning resources and focus on process supervision to effectively combine the rational development of resources, comprehensive utilization of energy and mining, and regional ecological environmental protection.

Second, the proportion of the tertiary sector and fiscal expenditures should be increased. The economic development level of the Loess Plateau still has a large gap with the eastern part of China, and the study found that the proportion of tertiary industry and the financial expenditure on science and technology have an important role in pulling the carrying capacity of the regional economic subsystem. Tongchuan has the highest growth rate of economic subsystem carrying capacity in the Loess Plateau region owing to the vigorous development of tourism in recent years, and Zhongwei has a faster growth rate of economic subsystem carrying capacity because of the significant increase in financial expenditure on science and technology in recent years. The cities with low RECC at present, such as Lvliang, Xinzhou, Baiyin, Tianshui, and Qingyang, can accelerate economic restructuring according to their characteristics, vigorously develop new industries, and accelerate the pulling of the regional economy to promote the improvement of RECC.

Third, for cities with better RECC at present, such as Ordos and the provincial capitals Taiyuan, Yinchuan, Lanzhou, and Xining, attention should be paid to the problems of duplicate construction, sloppy utilization, and idleness in the development process. The combination of ecological and environmental management, and economic development should be strengthened to avoid the phenomenon of duplicate construction and waste of resources.

Fourth, focus on the coordinated development of the Loess Plateau. The cities should take advantage of local conditions, actively participate in regional economic cooperation, and undertake foreign investment and industrial transfer. This can also gradually narrow the gap between urban and rural development using region-driven regions, and more finances can be invested in people’s livelihood, i.e., toward infrastructure, education, medical care, social security, and other aspects directly related to the interests of the masses.

## Supporting information

S1 Table Obstacle indicators for cities with positive RECC growth from 2013 to 2018 (top five). (DOCX)

S1 Data (XLSX)
