# Supplementary material for: Temporal and spatial evolution and obstacle diagnosis of resource and environment carrying capacity in the Loess Plateau
Source: PLoS One. 2021 Aug 18;16(8):e0256334. doi: 10.1371/journal.pone.0256334 (PMC8372938; doi:10.1371/journal.pone.0256334)
Supplement: S1 Table — (DOCX) [file pone.0256334.s001.docx]

**S1 Table. Obstacle indicators for cities with positive RECC growth from 2013 to 2018 (top five).**

| Year | | | 2013 | 2014 | 2015 | 2016 | 2017 | 2018 |
| --- | --- | --- | --- | --- | --- | --- | --- | --- |
| items | | | obstacle indicators / obstacle degree | | | | | |
| Obstacle ranking | Luoyang | 1 | X_3_/11.71 | X_3_/12.59 | X_10_/13.52 | X_3_/18.24 | X_3_/19.3 | X_13_/32.57 |
|  |  | 2 | X_10_/10.97 | X_10_/12.09 | X_3_/12.92 | X_10_/14.52 | X_10_/17.65 | X_8_/30.16 |
|  |  | 3 | X_19_/7.41 | X_19_/8.87 | X_19_/10.45 | X_11_/10.14 | X_7_/13.16 | X_11_/18.18 |
|  |  | 4 | X_12_/7.29 | X_12_/7.98 | X_12_/7.81 | X_7_/8.13 | X_13_/12.69 | X_15_/7.85 |
|  |  | 5 | X_11_/6.61 | X_14_/7.34 | X_1_/4.76 | X_12_/7.79 | X_8_/9.96 | X_20_/6.21 |
|  | Xining | 1 | X_14_/9.65 | X_14_/10.07 | X_14_/10.19 | X_13_/12.04 | X_3_/19.52 | X_13_/33.09 |
|  |  | 2 | X_21_/7.41 | X_19_/7.23 | X_21_/8.68 | X_3_/11.56 | X_13_/17.64 | X_8_/19.60 |
|  |  | 3 | X_12_/7.20 | X_12_/7.19 | X_11_/7.48 | X_14_/11.19 | X_16_/10.18 | X_16_/14.29 |
|  |  | 4 | X_2_/7.03 | X_7_/7.09 | X_6_/7.09 | X_16_/8.21 | X_4_/9.34 | X_7_/12.15 |
|  |  | 5 | X_6_/6.91 | X_2_/7.02 | X_12_/6.85 | X_11_/7.78 | X_8_/9.34 | X_21_/5.54 |
|  | Sanmenxia | 1 | X_14_/11.85 | X_14_/13.34 | X_14_/13.76 | X_11_/13.91 | X_21_/14.87 | X_21_/24.58 |
|  |  | 2 | X_18_/9.64 | X_18_/9.84 | X_18_/10.80 | X_13_/9.87 | X_13_/14.39 | X_11_/20.98 |
|  |  | 3 | X_15_/8.47 | X_15_/8.94 | X_15_/8.65 | X_3_/9.60 | X_16_/12.85 | X_8_/16.95 |
|  |  | 4 | X_20_/7.65 | X_13_/8.72 | X_20_/8.16 | X_10_/7.62 | X_8_/8.90 | X_9_/12.15 |
|  |  | 5 | X_19_/7.48 | X_19_/8.18 | X_16_/6.80 | X_18_/6.97 | X_7_/8.32 | X_15_/7.87 |
|  | Wuzhong | 1 | X_3_/15.19 | X_3_/15.93 | X_3_/16.43 | X_3_/21.72 | X_4_/23.93 | X_4_/31.56 |
|  |  | 2 | X_4_/11.17 | X_4_/9.09 | X_4_/14.84 | X_16_/15.09 | X_3_/19.09 | X_16_/21.95 |
|  |  | 3 | X_9_/6.37 | X_7_/6.83 | X_16_/10.04 | X_7_/12.38 | X_8_/10.31 | X_8_/18.70 |
|  |  | 4 | X_10_/6.19 | X_16_/6.33 | X_12_/6.68 | X_10_/7.73 | X_7_/10.24 | X_13_/12.74 |
|  |  | 5 | X_14_/6.09 | X_14_/5.62 | X_7_/6.44 | X_8_/7.27 | X_16_/7.36 | X_5_/4.65 |
|  | Lanzhou | 1 | X_5_/10.39 | X_5_/12.10 | X_11_/12.18 | X_7_/18.24 | X_5_/21.98 | X_5_/33.32 |
|  |  | 2 | X_19_/7.32 | X_9_/8.38 | X_7_/11.14 | X_9_/15.42 | X_7_/19.67 | X_8_/26.54 |
|  |  | 3 | X_9_/6.71 | X_11_/7.61 | X_19_/8.39 | X_11_/13.98 | X_8_/14.72 | X_13_/14.84 |
|  |  | 4 | X_7_/6.71 | X_7_/7.31 | X_15_/7.26 | X_8_/10.06 | X_11_/10.44 | X_3_/11.88 |
|  |  | 5 | X_15_/6.51 | X_3_/7.22 | X_21_/6.67 | X_3_/8.07 | X_16_/6.61 | X_9_/4.81 |
|  | Zhongwei | 1 | X_20_/13.86 | X_20_/14.80 | X_17_/13.24 | X_17_/17.02 | X_14_/21.00 | X_14_/23.53 |
|  |  | 2 | X_9_/9.57 | X_17_/12.10 | X_20_/12.71 | X_20_/14.63 | X_17_/19.56 | X_13_/16.83 |
|  |  | 3 | X_19_/8.16 | X_9_/10.27 | X_14_/11.85 | X_13_/10.98 | X_13_/10.15 | X_17_/12.75 |
|  |  | 4 | X_21_/7.98 | X_2_/8.44 | X_9_/7.37 | X_9_/6.30 | X_18_/8.60 | X_8_/11.40 |
|  |  | 5 | X_7_/6.62 | X_19_/8.39 | X_2_/7.05 | X_21_/5.86 | X_8_/7.36 | X_7_/10.70 |
|  | Jinzhong | 1 | X_1_/13.32 | X_1_/14.39 | X_1_/16.24 | X_1_/20.06 | X_4_/14.90 | X_8_/31.68 |
|  |  | 2 | X_18_/9.22 | X_18_/10.66 | X_4_/12.11 | X_4_/16.67 | X_8_/14.13 | X_13_/23.84 |
|  |  | 3 | X_19_/8.79 | X_19_/9.38 | X_18_/10.41 | X_3_/7.11 | X_1_/12.33 | X_7_/12.09 |
|  |  | 4 | X_14_/7.98 | X_14_/9.05 | X_19_/9.53 | X_12_/6.53 | X_21_/11.66 | X_3_/11.97 |
|  |  | 5 | X_4_/6.41 | X_4_/6.79 | X_14_/6.33 | X_18_/6.43 | X_13_/7.92 | X_21_/8.21 |
|  | Yinchuan | 1 | X_21_/15.56 | X_21_/14.13 | X_21_/18.08 | X_2_/15.36 | X_21_/29.40 | X_21_/34.61 |
|  |  | 2 | X_11_/10.49 | X_11_/11.43 | X_11_/11.87 | X_17_/10.92 | X_2_/10.82 | X_8_/12.66 |
|  |  | 3 | X_2_/9.87 | X_2_/10.81 | X_2_/10.60 | X_3_/10.38 | X_11_/10.37 | X_16_/11.40 |
|  |  | 4 | X_10_/7.99 | X_10_/8.82 | X_10_/9.91 | X_7_/9.27 | X_7_/9.23 | X_17_/9.96 |
|  |  | 5 | X_9_/5.55 | X_17_/7.06 | X_3_/7.01 | X_6_/8.65 | X_8_/9.13 | X_5_/8.65 |
| Obstacle ranking | Baiyin | 1 | X_10_/18.07 | X_10_/21.17 | X_10_/21.41 | X_10_/27.30 | X_7_/23.24 | X_10_/59.88 |
|  |  | 2 | X_7_/11.89 | X_7_/13.91 | X_7_/14.51 | X_7_/17.79 | X_13_/21.70 | X_5_/11.80 |
|  |  | 3 | X_13_/9.76 | X_13_/10.40 | X_1_/10.54 | X_13_/12.88 | X_1_/18.40 | X_3_/11.73 |
|  |  | 4 | X_1_/9.13 | X_1_/8.44 | X_13_/10.17 | X_1_/11.95 | X_11_/9.31 | X_8_/7.37 |
|  |  | 5 | X_18_/4.63 | X_12_/5.51 | X_11_/5.80 | X_11_/6.14 | X_5_/6.95 | X_18_/4.10 |
|  | Datong | 1 | X_1_/9.58 | X_9_/10.94 | X_9_/10.78 | X_1_/11.77 | X_13_/13.31 | X_8_/25.38 |
|  |  | 2 | X_9_/9.41 | X_1_/9.29 | X_1_/8.34 | X_12_/11.26 | X_12_/11.72 | X_12_/21.67 |
|  |  | 3 | X_5_/8.44 | X_10_/9.19 | X_3_/8.13 | X_4_/11.06 | X_8_/10.98 | X_13_/12.40 |
|  |  | 4 | X_10_/8.36 | X_5_/8.43 | X_4_/7.90 | X_3_/8.37 | X_7_/10.73 | X_11_/9.01 |
|  |  | 5 | X_19_/7.75 | X_19_/7.45 | X_5_/7.57 | X_13_/7.7 | X_1_/10.35 | X_16_/8.60 |
|  | Tongchuan | 1 | X_19_/10.77 | X_19_/11.23 | X_19_/12.91 | X_8_/14.40 | X_7_/13.90 | X_21_/27.25 |
|  |  | 2 | X_17_/9.45 | X_17_/10.29 | X_17_/11.77 | X_7_/12.44 | X_10_/13.66 | X_18_/26.73 |
|  |  | 3 | X_8_/9.10 | X_8_/10.02 | X_8_/11.77 | X_10_/11.20 | X_8_/13.42 | X_16_/14.91 |
|  |  | 4 | X_7_/6.89 | X_7_/8.55 | X_10_/9.28 | X_1_/9.04 | X_21_/11.47 | X_14_/14.69 |
|  |  | 5 | X_20_/6.53 | X_13_/6.92 | X_14_/7.63 | X_13_/7.58 | X_4_/8.78 | X_1_/6.36 |
|  | Taiyuan | 1 | X_12_/8.83 | X_12_/11.58 | X_21_/10.65 | X_12_/11.88 | X_5_/9.72 | X_8_/17.81 |
|  |  | 2 | X_4_/8.15 | X_4_/9.79 | X_11_/10.26 | X_4_/11.10 | X_4_/9.67 | X_7_/16.64 |
|  |  | 3 | X_1_/7.85 | X_1_/9.55 | X_4_/9.93 | X_21_/10.22 | X_16_/9.65 | X_13_/14.92 |
|  |  | 4 | X_21_/7.52 | X_5_/9.20 | X_1_/9.51 | X_5_/9.33 | X_21_/9.59 | X_12_/13.23 |
|  |  | 5 | X_5_/7.49 | X_19_/8.85 | X_12_/8.15 | X_1_/9.29 | X_8_/7.65 | X_11_/12.37 |
|  | Shuozhou | 1 | X_15_/13.81 | X_9_/10.23 | X_13_/11.44 | X_13_/11.62 | X_13_/11.88 | X_13_/20.57 |
|  |  | 2 | X_9_/12.36 | X_10_/10.04 | X_15_/9.91 | X_3_/9.56 | X_14_/11.64 | X_3_/16.11 |
|  |  | 3 | X_10_/11.33 | X_15_/9.97 | X_18_/7.79 | X_14_/8.92 | X_5_/9.45 | X_8_/11.43 |
|  |  | 4 | X_7_/10.87 | X_7_/8.98 | X_11_/7.46 | X_5_/7.84 | X_3_/8.13 | X_14_/11.05 |
|  |  | 5 | X_19_/9.52 | X_18_/8.28 | X_9_/7.39 | X_20_/7.50 | X_16_/7.94 | X_5_/8.06 |
|  | Baotou | 1 | X_15_/10.85 | X_15_/12.18 | X_15_/15.60 | X_15_/14.44 | X_4_/12.32 | X_16_/21.23 |
|  |  | 2 | X_9_/9.04 | X_9_/11.42 | X_9_/11.11 | X_16_/12.80 | X_8_/12.09 | X_8_/18.99 |
|  |  | 3 | X_19_/8.14 | X_19_/9.95 | X_19_/9.81 | X_9_/10.19 | X_16_/11.22 | X_4_/17.02 |
|  |  | 4 | X_17_/7.50 | X_17_/9.63 | X_20_/8.43 | X_8_/9.75 | X_3_/9.39 | X_14_/11.60 |
|  |  | 5 | X_20_/7.14 | X_20_/8.12 | X_1_/8.04 | X_1_/8.37 | X_7_/8.42 | X_3_/10.74 |
|  | Bayannaoer | 1 | X_11_/18.00 | X_11_/21.01 | X_11_/23.10 | X_11_/25.19 | X_11_/28.91 | X_16_/14.95 |
|  |  | 2 | X_20_/9.68 | X_20_/10.99 | X_19_/9.16 | X_16_/17.82 | X_16_/16.16 | X_4_/13.20 |
|  |  | 3 | X_19_/9.00 | X_19_/9.99 | X_7_/6.88 | X_7_/10.74 | X_4_/10.48 | X_20_/12.25 |
|  |  | 4 | X_2_/7.59 | X_2_/7.96 | X_18_/6.74 | X_9_/6.90 | X_1_/9.69 | X_8_/10.70 |
|  |  | 5 | X_9_/6.21 | X_3_/7.15 | X_3_/6.53 | X_2_/6.30 | X_8_/8.70 | X_5_/8.71 |
|  | Dingxi | 1 | X_18_/10.03 | X_18_/13.29 | X_21_/13.24 | X_3_/17.66 | X_8_/15.77 | X_8_/26.36 |
|  |  | 2 | X_20_/8.42 | X_8_/10.11 | X_8_/11.41 | X_8_/14.80 | X_3_/13.54 | X_3_/20.62 |
|  |  | 3 | X_6_/8.03 | X_20_/9.47 | X_3_/11.32 | X_21_/13.68 | X_5_/12.70 | X_5_/15.55 |
|  |  | 4 | X_2_/7.80 | X_2_/8.27 | X_18_/9.97 | X_11_/12.87 | X_21_/10.71 | X_13_/10.08 |
|  |  | 5 | X_9_/7.50 | X_6_/8.01 | X_11_/8.56 | X_18_/6.53 | X_16_/8.77 | X_21_/8.63 |
|  | Yulin | 1 | X_19_/14.23 | X_19_/12.97 | X_14_/15.22 | X_12_/17.17 | X_14_/21.21 | X_14_/24.86 |
|  |  | 2 | X_1_/10.04 | X_12_/12.65 | X_1_/9.92 | X_14_/14.69 | X_12_/10.97 | X_12_/14.85 |
|  |  | 3 | X_6_/9.33 | X_1_/8.75 | X_19_/8.95 | X_15_/14.42 | X_16_/10.77 | X_16_/14.17 |
| Obstacle ranking |  | 4 | X_9_/9.19 | X_4_/7.51 | X_15_/8.83 | X_1_/13.23 | X_15_/9.11 | X_8_/10.82 |
|  |  | 5 | X_4_/8.46 | X_6_/7.20 | X_12_/7.62 | X_4_/14.47 | X_7_/8.39 | X_15_/10.06 |
|  | Qingyang | 1 | X_7_/22.10 | X_14_/23.08 | X_14_/22.83 | X_14_/25.72 | X_14_/29.56 | X_14_/42.21 |
|  |  | 2 | X_10_/12.99 | X_7_/18.21 | X_7_/18.48 | X_7_/16.37 | X_7_/17.05 | X_3_/14.22 |
|  |  | 3 | X_9_/9.70 | X_10_/11.39 | X_10_/12.11 | X_10_/10.94 | X_10_/6.66 | X_5_/9.87 |
|  |  | 4 | X_2_/8.32 | X_9_/7.34 | X_11_/7.07 | X_3_/8.50 | X_4_/6.07 | X_8_/9.30 |
|  |  | 5 | X_6_/6.51 | X_17_/6.49 | X_9_/6.08 | X_11_/6.67 | X_5_/5.80 | X_16_/9.23 |
|  | Guyuan | 1 | X_20_/12.44 | X_20_/13.70 | X_20_/13.43 | X_14_/14.47 | X_14_/20.14 | X_16_/19.58 |
|  |  | 2 | X_14_/9.76 | X_14_/10.39 | X_3_/12.37 | X_3_/13.18 | X_16_/14.88 | X_8_/16.03 |
|  |  | 3 | X_9_/9.21 | X_9_/9.60 | X_17_/10.94 | X_20_/12.42 | X_3_/14.34 | X_7_/13.97 |
|  |  | 4 | X_17_/8.71 | X_17_/7.10 | X_9_/9.02 | X_11_/9.15 | X_20_/11.29 | X_14_/12.74 |
|  |  | 5 | X_12_/7.42 | X_12_/7.01 | X_11_/7.37 | X_17_/7.60 | X_11_/7.37 | X_13_/9.62 |
|  | Xinzhou | 1 | X_1_/12.91 | X_18_/13.18 | X_18_/13.44 | X_18_/14.88 | X_8_/14.48 | X_3_/31.82 |
|  |  | 2 | X_18_/11.86 | X_3_/12.68 | X_3_/12.74 | X_3_/13.31 | X_3_/11.72 | X_8_/20.02 |
|  |  | 3 | X_19_/8.27 | X_1_/11.33 | X_1_/12.34 | X_1_/12.48 | X_9_/11.18 | X_16_/17.45 |
|  |  | 4 | X_9_/7.98 | X_19_/7.88 | X_19_/11.18 | X_19_/11.94 | X_16_/9.40 | X_13_/12.84 |
|  |  | 5 | X_7_/7.18 | X_9_/6.97 | X_11_/7.06 | X_4_/7.13 | X_1_/9.20 | X_11_/5.90 |
